# Preparation of a PEGylated liposome that co-encapsulates l-arginine and doxorubicin to achieve a synergistic anticancer effect†

**DOI:** 10.1039/d1ra06514a

**Published:** 2021-10-21

**Authors:** Haitao Feng, Jeong-Hun Kang, Song Qi, Akihiro Kishimura, Takeshi Mori, Yoshiki Katayama

**Affiliations:** Department of Applied Chemistry, Graduate School of Engineering, Kyushu University 744 Motooka, Nishi-ku Fukuoka 819-0395 Japan; Division of Biopharmaceutics and Pharmacokinetics, National Cerebral and Cardiovascular Center Research Institute 6-1 Shinmachi, Kishibe Suita Osaka 564-8565 Japan; Graduate School of Systems Life Sciences, Kyushu University 744 Motooka, Nishi-ku Fukuoka 819-0395 Japan; Center for Future Chemistry, Kyushu University 744 Motooka, Nishi-ku Fukuoka 819-0395 Japan; International Research Center for Molecular Systems, Kyushu University 744 Motooka, Nishi-ku Fukuoka 819-0395 Japan; Centre for Advanced Medicine Innovation, Kyushu University 3-1-1 Maidashi, Higashi-ku Fukuoka 812-8582 Japan; Department of Biomedical Engineering, Chung Yuan Christian University 200 Chung Pei Rd., Chung Li 32023 ROC Taiwan

## Abstract

Strategies that combine chemotherapies with unconventional agents such as nitric oxide (NO) have been shown to enhance cancer therapies. Compared with small molecule chemotherapy drugs, nanosized particles have improved therapeutic efficacies and reduced systemic side effects because of the enhanced permeability and retention effect. In this report, we prepared PEGylated liposomes (LP) that incorporated l-arginine (Arg) and the anticancer drug doxorubicin (Dox) to yield a co-delivery system (Dox–Arg-LP). On the basis of our previous research, we hypothesized that Dox–Arg-LP should achieve a synergistic anticancer effect because Arg conversion to NO by activated M1 macrophages augments the chemotherapeutic activity of Dox. Dox–Arg-LP showed comparable physical properties to those of conventional Dox-only liposomes (Dox-LP). *In vitro* assessment revealed that the cytotoxicity of Dox–Arg-LP toward cancer cells was significantly higher than that of Dox-LP. *In vivo* application of Dox–Arg-LP in mice enhanced the chemotherapeutic effect with a 2 mg kg^−1^ dose of Dox–Arg-LP achieving the same therapeutic efficacy as a two-fold higher dose of Dox-LP (*i.e.*, 4 mg kg^−1^). Therefore, co-encapsulation of dual agents into a liposome formulation is an efficient strategy to enhance chemotherapy while reducing systemic toxicity.

## Abstract 2

Strategies that combine chemotherapies with unconventional agents such as nitric oxide (NO) have been shown to enhance cancer therapies.

## Graphical abstract

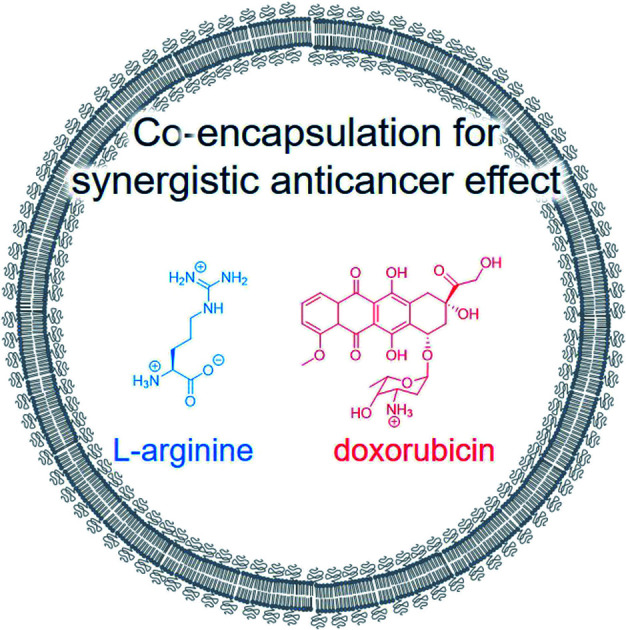

## Introduction

Various remarkable cancer therapies have been developed with chemotherapies still playing a leading role in cancer treatment. Doxorubicin (Dox) is a well-known first-line chemotherapeutic drug used to treat several types of human cancers, and a nanoscale formulation of doxorubiciikn (Doxil) was approved for clinical use by the US Food and Drug Administration more than 20 years ago. Doxil has an improved safety profile compared with that of the free form. Nano-formulations can passively accumulate in solid tumors because of the enhanced permeability and retention (EPR) effect, thereby leading to tumor drug concentration increases of up to 5-fold compared with that of the free drug.^1^ However, in clinical settings, unsatisfactory effectiveness and some severe side effects still limit the application of Doxil. Thus, development of a strategy with efficient response and reduced side effects is urgently needed for treating cancer patients with Doxil.

Combination therapy has recently been acknowledged in cancer management as an approach to circumvent the limitation of low efficiency caused by a single therapeutic, which can lead to tumor recurrence and metastasis.^2–4^ Previous efforts have validated the benefits of combining chemotherapies, such as the combination of oxaliplatin, leucovorin, 5-fluorouracil and irinotecan to treat pancreatic cancer,^5^ or paclitaxel and carboplatin to treat ovarian cancer.^6^ Nevertheless, drawbacks including poor aqueous solubility of the drugs, a narrow therapeutic index and severe side effects have limited the efficiency response in cancer patients.^7,8^

Therefore, promising different therapeutic mechanisms with potential advantages (*e.g.*, synergistic effects) that cooperatively suppress cancer progression are being pursued.^9–11^ In the tumor microenvironment, an essential component that constitutes a large percentage (up to 50%) of the tumor mass is tumor associated macrophages (TAM).^12,13^ Recently, TAM have been found to be a promising target for inducing tumor cytotoxicity. TAM can be classified into two phenotypes, M1 and M2, based on the stage of cancer development. M1 is the main phenotype during the early stages of cancer progression. This TAM phenotype expresses a high level of inducible nitric oxide synthase (iNOS), which catalyzes the conversion of l-arginine (Arg) to soluble nitric oxide (NO). Secreted NO displays antitumor activity through various mechanisms, such as stimulating the production of cytotoxic reactive nitrogen species, inducing cell shrinkage, causing extensive DNA damage and inhibiting anti-apoptotic survival signaling.^14–17^ We recently showed that supplementing Dox with Arg afforded a synergistic anticancer effect with the help of activated M1 macrophages *in vitro*.^18^ Hence, in the present study, this synergistic effect was further exploited by co-encapsulating Arg and Dox in PEGylated liposomes (Dox–Arg-LP) to utilize the EPR effect. Arg was incorporated into the liposome using a hydration procedure and Dox was then efficiently encapsulated into the liposome through a remote loading strategy. We hypothesized that Dox–Arg-LP should give a synergistic and enhanced anticancer effect compared with that of liposomes loaded with only Dox (Dox-LP). Firstly, because of the EPR effect, Dox–Arg-LP could be highly delivered and distributed to the tumor tissues. Moreover, because TAM can accumulate large drug deposits,^19^ we show that TAM accumulate high amounts of LP and release their cytotoxic payload to the surrounding tumor tissue as a chemotherapy. Arg acted as a substrate for iNOS in M1 macrophages, which led to the production of cytotoxic NO. This increase in NO levels enhanced the vulnerability and sensitivity of cancer cells towards released Dox, thereby affording a synergistic effect that enhanced antitumor efficiency while reducing systemic toxicity. Therefore, Dox–Arg-LP is a promising approach that can be used to augment anticancer nanomedicines to give specific, enhanced therapeutic effects.

## Results and discussion

### Preparation of Arg-containing liposomes (Arg-LP)

Liposomes are typically used for effective delivery of medicines. Arg is a water-soluble amino acid that was incorporated into PEGylated liposomes through the hydration process. The encapsulation efficiency of Arg was quantified using the K-LARGE kit and was found to be around 3%. The obtained liposomes were suspended in neutral buffer at 4 °C, which is well below the gel-liquid crystalline phase transition temperature of the matrix lipid, HSPC (53 °C).^20^ The characteristics of the liposomes are presented in Table 1. The diameters of the liposomes prepared are a suitable size (less than 200 nm) for accumulation in tumors *via* the EPR effect.^21^

**Table 1:** Characteristics of the prepared liposomes^a^

Liposome	Mean diameter ± SEM (nm)	PDI	*ζ*-potential ± SEM (mV)	EE%
LP	105 ± 1.2	0.05	−19 ± 0.5	—
Arg-LP	101 ± 1.2	0.05	−18 ± 0.5	—
Dox-LP	118 ± 1.7	0.09	−19 ± 0.9	83
Dox–Arg-Lp	116 ± 1.9	0.10	−18 ± 0.5	73

a PDI, polydispersity index; EE, encapsulation efficiency.

### NO production by macrophages incubated with Arg-LP

**Fig. 1:**
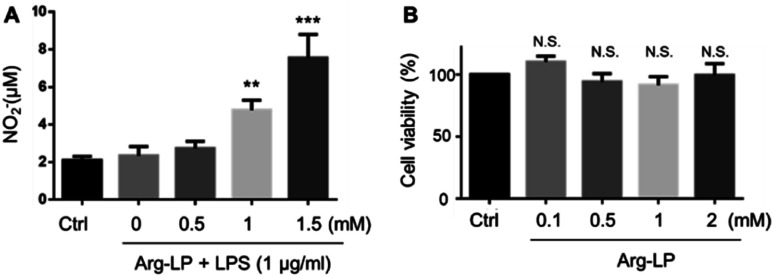
(A) NO production by RAW 264.7 macrophages incubated with different amounts of Arg-LP, as defined by the Arg concentration. After 6 h incubation with LPS (1 μg mL^−1^), Arg-LP was added to the cells and incubated for a further 48 h. (B) The cytotoxicity of Arg-LP to macrophages after 48 h incubation. Data are expressed as mean ± SD (*n* = 3). **, *p* < 0.01; ***, *p* < 0.001 compared with the control. N. S., not significant.

NO production by macrophages using Arg as the substrate in response to lipopolysaccharides (LPS) stimulation has been reported.^18,22^ Here, we evaluated NO production by macrophages incubated with Arg-LP. Macrophages were first stimulated with LPS (1 μg mL^−1^) for 6 h to activate the expression of iNOS and then incubated with different amounts (defined as Arg concentration in mM) of Arg-LP for 48 h. The produced NO was detected by Griese reagent. As shown in Fig. 1A, Arg incorporated into liposomes induced NO production by activated macrophages in a concentration dependent manner. Thus, Arg-LP is a promising substrate source for production of NO by stimulated TAM. The cytotoxicity of the Arg-LP was also examined. Different amounts of Arg-LP, as defined by the Arg concentration, were incubated with macrophages for 48 h. The Cell Counting Kit-8 reagent was used to evaluate cell viability. No significant toxicity up to 2 mM Arg-containing LP was observed (Fig. 1B), which indicates that macrophage viability was not affected by Arg-LP.

### Preparation of Dox–Arg-LP

Following the successful preparation of Arg-LP, we sought to encapsulate Dox into Arg containing liposomes by using a remote loading method to yield an efficient drug delivery system with reduced side effects. Based on our previous studies,^18,23^ we hypothesized that combining Arg and Dox in a single delivery system should afford an efficient synergistic anticancer effect. Remote loading of Dox into liposomes was performed by using a pH gradient between the internal and external solutions. The physical characteristics of the prepared LP are summarized in Table 1. The diameter, polydispersity index (PDI) and EE% of the prepared liposomes were very similar (Table 1). As shown for introducing Arg into LP, the EE% of Dox is modest, which indicates that incorporation of Arg into conventional Dox-LP is attainable.

**Fig. 2:**
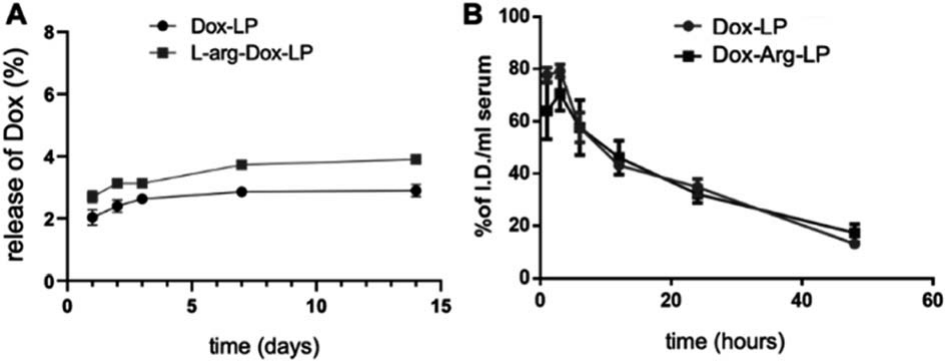
(A) Dox release profiles of the prepared LP at pH 7.4 and 4 °C. Dox release was quantified by UV absorption at 495 nm. (B) Changes in the liposome concentration in mouse serum after intravenous injection into mice. Data are expressed as mean ± SD (*n* = 3).

The stability and drug release rate of the prepared Dox–Arg-LP and Dox-LP were compared by first storing the samples at 4 °C for the indicated time and then examining the drug release rate and basic characteristics, including size and PDI (Fig. 2A and S2†). Arg incorporated into the liposomes did not affect the retention of incorporated Dox and colloidal stability. The blood circulation half-lives of Dox–Arg-LP and Dox-LP were evaluated after intravenous administration into BALB/c mice. The blood clearance profile of Dox–Arg-LP was comparable to that of Dox-LP (Fig. 2B). The blood half-life was estimated to be ∼12 h for both liposomes, which is in agreement with previous half-lives reported for PEGylated liposomes in mice.^24^ The results showed that co-incorporation of Arg and Dox into liposomes was feasible, and this Dox–Arg-LP is a promising nanoparticle that is a new modality for enhancing anti-cancer therapy.

### Dox–Arg-LP augments the cytotoxicity of cancer cells

**Fig. 3:**
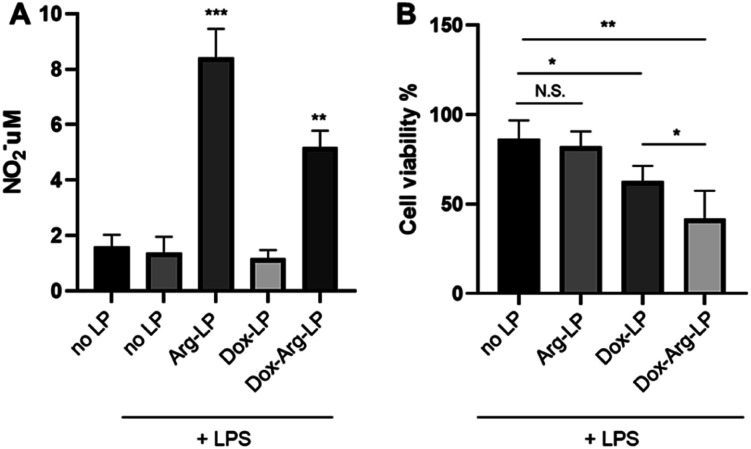
(A) NO production by RAW 264.7 macrophages incubated with liposomes loaded with different compounds. After 6 h incubation with LPS (1 μg mL^−1^), liposomes were added to the cells and incubated for another 48 h (Dox, 1 μM; Arg, 1 mM). (B) Synergistic effect of Dox–Arg-LP on the cytotoxicity of cancer cells. LP samples were added to the upper chamber and incubated for 48 h. The results are expressed as mean ± SD (*n* = 3). *, *p* < 0.05; **, *p* < 0.01; ***, *p* < 0.001; N. S., not significant. As for panel A, the *t*-test was conducted by comparison with no LP + LPS.

As mentioned above, our previous work showed that introducing Arg augments the cytotoxicity of Dox toward cancer cells. Herein, we evaluated whether this augmentation phenomenon exists when co-encapsulating Arg and Dox into liposomes. A previous protocol that mimics the tumor environment was used^25^ with a co-culture system. Here, cancer cells were cultured in the lower chamber while LP were introduced to the upper chamber seeded with LPS-activated macrophages. As shown in Fig. 3A, co-encapsulation of Arg and Dox in LP gave a clear increase in NO production by macrophages compared with NO production by macrophages incubated with Dox-LP. Conversely, the level of NO production by macrophages incubated with Dox–Arg-LP was lower compared with macrophages incubated with Arg-LP, indicating a modest level of Dox-induced cytotoxicity. Nonetheless, as Fig. 3B showed, treatment with Dox–Arg-LP reduced the viability of cancer cells ∼20% more effectively than Dox-LP because of the synergistic effect of Dox and NO secreted by macrophages.^26^ As showed in Fig. S1,† lower sensitivity of macrophages than cancer cells toward Dox enabled NO production and Dox release by macrophages after uptake of Dox–Arg-LP.

Research has shown that in the tumor microenvironment macrophages act as a reservoir for the release of nanoparticles. Thus, macrophages play an essential role in this form of cancer treatment by phagocytosing nanoparticles and then gradually releasing the payload to the surrounding tumor cells.^19,25^ In the present study, free diffusion of LP through the chamber membrane was inhibited because of the high density of macrophages cultured in the upper chamber.^25^ The lower cell viability of cancer cells in the coculture system incubated with Dox-LP compared with the no LP control is attributed to the substantial capture of Dox-LP by macrophages, followed by the release of Dox and subsequent uptake by cancer cells. In contrast, NO produced by macrophages that phagocytosed Arg-LP only slightly affected cancer cell viability (Fig. 3B). However, a significant reduction in cell viability was observed when Dox–Arg-LP were introduced to the macrophages, which indicates that conversion of Arg by iNOS to NO increased the sensitivity of cancer cells toward Dox and this phenomenon was reserved by incorporating both compounds into liposomes.

### Dox–Arg-LP shows an enhanced antitumor effect

**Fig. 4:**
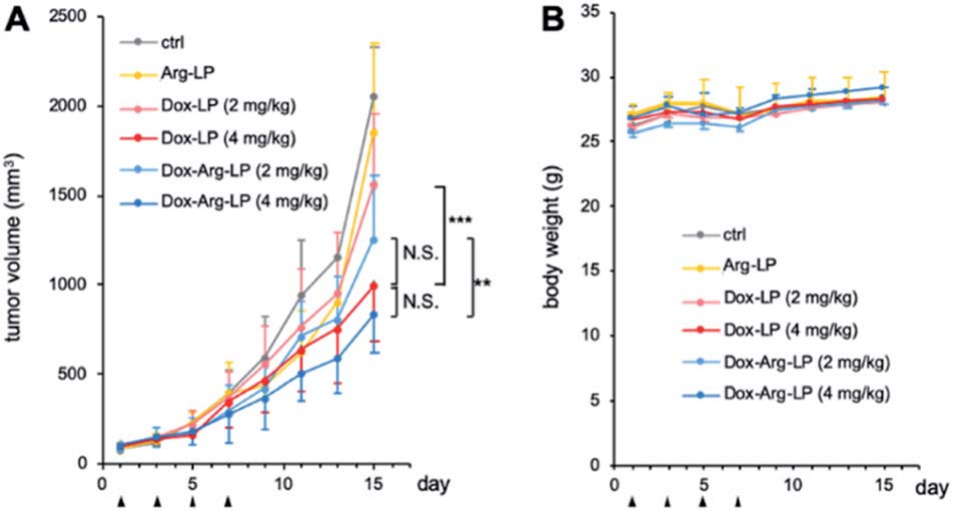
(A) Therapeutic efficacy of Dox–Arg-LP and (B) change in body weight during treatment. Mice were intravenously injected with various types of LP at day 1, 3, 5 and 7 (indicated by the arrows). Results are expressed as means ± SD (*n* = 6). **, *p* < 0.01; ***, *p* < 0.001 compared with the control.

LP samples were intravenously injected into CT26-xenografted mice to investigate the synergistic anticancer potency of the drug-loaded liposomes. Mice with tumor volumes of ∼100 mm^3^ were randomly assigned into six groups (*n* = 6). Dox-LP and Dox–Arg-LP were injected into mice at a Dox dosage equivalent of 2 or 4 mg kg^−1^. Intravenous administration of PBS and Arg-LP were also evaluated as controls. The tumor volume was monitored to determine the antitumor effect and this monitoring was continued until the diameter of the tumor was over 20 mm. Growth of tumors increased rapidly for groups treated with PBS or Arg-LP (Fig. 4A), which indicates that Arg cannot inhibit the growth of cancer cells, which is consistent with the *in vitro* results. In contrast, the rate of tumor growth in mice treated with Dox-LP (Dox 4 mg kg^−1^) or Dox–Arg-LP (Dox 2 mg kg^−1^ and 4 mg kg^−1^) was reduced compared with the other groups. Among all groups, mice treated with Dox-LP (4 mg kg^−1^) and Dox–Arg-LP (4 mg kg^−1^) displayed the best prevention efficacy of tumor growth 15 days post the first injection. Although there is no significant difference between these two groups, Dox–Arg-LP (4 mg kg^−1^) showed the strongest tendency to prevent tumor growth, which indicates that further optimization of the dosage and treatment procedure should afford stronger inhibition of tumor growth. Additionally, no significant difference was observed between the Dox-LP (4 mg kg^−1^) and Dox–Arg-LP (2 mg kg^−1^) treatment group. This observation indicates that half the Dox dosage and co-encapsulating with Arg yields the same therapeutic effect as double the Dox dosage; thus, Dox-induced toxic side effects can be reduced by lowering the Dox dosage. This synergistic antitumor effect of Dox and Arg in mice corroborates the *in vitro* results. The enhanced efficacy of the Dox–Arg-LP can possible be ascribed to conversion of Arg to NO by macrophages, which increased the sensitivity of cancer cells to Dox. No noticeable changes in body weight were observed among the different treatment groups (Fig. 4B), which indicates that Dox–Arg-LP has no systemic toxicity. Therefore, using liposomes to co-deliver Arg and Dox represents a promising strategy for efficient and safe cancer treatment.

## Experimental

### Materials

Hydrogenated soybean phosphatidylcholine (HSPC) and *N*-(carbonyl-methoxypolyethyleneglycol2000)-1,2-distearoyl-*snglycero*-3-phosphoethanolamine (DSPE-PEG) were purchased from NOF Corporation (Tokyo, Japan). Cholesterol was obtained from Tokyo Chemical Industry Co., Ltd (Tokyo, Japan). 1,1′-Dioctadecyltetramethyl indotricarbocyanine iodide (XenoLight DiR) was supplied by Caliper Life Sciences (Hopkinton, MA, USA). l-Arginine hydrochloride, doxorubicin, lipopolysaccharides (LPS) from *Escherichia coli* were purchased from Sigma Chemical Co. (St. Louis, MO, USA). l-Arginine deficient SILAC Dulbecco's modified Eagle's medium (DMEM) was obtained from Thermo Scientific (Waltham, MA, USA).

### Cell line

RAW 264.7 macrophage and CT-26 murine colon adenocarcinoma cell were purchased from American Type Culture Collection (ATCC) and cultured in DMEM medium supplemented with 10% fetal bovine serum (FBS) (all were purchased from Gibco Invitrogen Co., Grand Island, NY, USA). The cells were maintained in a humidified incubator containing 5% CO_2_ in air at 37 °C.

### Mice

Male 6-week-old BALB/c mice were purchased from Kyudo. Co., Ltd (Saga, Japan). All animal procedures were performed in accordance with the Guidelines for Care and Use of Laboratory Animals of Kyushu University and approved by the Animal Ethics Committee of Kyushu University.

### Preparation of Dox–Arg-LP

LPs were prepared *via* the lipid-thin film hydration method as our previous work. Solutions of HSPC, cholesterol, and DSPE-PEG in chloroform were mixed in a molar ratio of 55 : 40 : 5, if needed, 1 mol% of Xenolight DiR (*vs.* total lipids) was added as fluorophore. Next, the chloroform was removed to create a lipid film using a rotary evaporator and a 55 °C water bath to maintain the temperature above the gel–liquid crystal transition temperature of HSPC (52 °C). The dried lipid film (100 μmol total lipid) was hydrated with 1 mL of 250 mM ammonium sulphate buffer (pH 5.5) alone or combined with 250 mM Arg, by intermittently heating and vortexing at 55 °C for 30 min. LPs were subsequently extruded through 200 nm (21 times) and then 50 nm polycarbonate membranes (41 times). The external solution was changed by ultracentrifugation (400 000×*g*) for 30 min at 4 °C with 10 mM DPBS (pH 7.4) buffer. The incorporated Arg was determined by a kit (K-LARGE Megazyme) which uses the principle of four step enzymatic consumption of NADPH. Then, the liposome solution was mixed with indicated concentrations of Dox, heated at 55 °C at water bath for 1 h to remote load the Dox into the liposome. The unencapsulated Dox was replaced by ultracentrifugation (400 000×*g*) for 30 min at 4 °C with DPBS buffer. The obtained liposome was stored at 4 °C. The average hydrodynamic diameter and *ζ* potential were determined using a Zetasizer Nano ZS ZEN3600 (Malvern Instruments Ltd, Worcestershire, UK).

### Drug encapsulation efficiency and the stability of Dox–Arg-LP

The encapsulation efficiency (EE%) was calculated using the formula below:EE% = (*W*_t_ − *W*_o_)/*W*_t_× 100%where *W*_t_ is the total amount of drug in the initial suspension and *W*_o_ is the quantity of drugs detected in the supernatant after ultracentrifugation to remove the liposomes. Concentrations of the drug in the supernatant were quantitatively analyzed using the spectrophotometer at the absorbance of 495 nm. To determine the storage stability of Dox–Arg-LP, the sample solution (pH7.4) was stored at 4 °C for up to two weeks. At each time point, the sample was treated as described above and calculated the released drug from the supernatant. The drug release rate was calculated using the formula:Drug release rate (%) = (*W*_o_/*W*_t_) × 100%

### Measurement of NO release by macrophages

RAW 264.7 macrophage cell line was seeded into 24-well plate (50 000 cells per well) and incubated for 24 h. After that, the medium was removed, and cells were incubated with fresh Arg deficient medium with or without the pre-stimulation of LPS (1 μg mL^−1^) for 6 h. Next, cells were incubated with various concentrations of Arg-containing LP for another 48 h. The supernatant of the cultured medium was collected and centrifugated at 1500×*g* for 15 min. Nitrite (NO_2_^−^), long thought to be a biologically inert product of nitric oxide (NO) oxidation, is recognized as a physiological NO storage pool. The amount of NO which reflected by nitrite was quantified by using the Griess Reagent Kit (Dojindo, Kumamoto, Japan) according to the manufacturer's instructions. The absorbance was measured at 540 nm using Infinite M Plex microplate reader (TECAN, Switzerland).

### Chemotherapy resistance assay

RAW 264.7 and CT26 colon carcinoma line were seeded into 96-well plate separately at the density of 5000 cells per well and treated with Dox at various concentrations (0.25, 0.5, 1, 2, 4, or 8 μM). After 48 h, Cell Counting kit 8 reagent (Dojindo, Kumamoto, Japan) was added into the cell and incubated for 4 h to detect the cell viability. The absorbance was measured at 450 nm by using Infinite M Plex microplate reader. Cell growth curve was accordingly charted, and the half maximal inhibitory concentration (IC_50_) was calculated using Probit regression analysis by GraphPad Prism software.

### Measurement of the cytotoxicity of Arg-LP

RAW 264.7 cells were seeded into 96-well plate (5000 cells per well) and incubated for 24 h. Then indicated concentrations of Arg-LP were added to the RAW 264.7 cell and incubated for another 48 h. After that, CCK-8 assay was performed as described above. The absorbance was measured at 450 nm by using Infinite M Plex microplate reader.

### Blood circulation stability

To compare the blood circulation stability of Dox–Arg-LP with the conventional Dox LP, 6-week-old BALB/c mice (*n* = 18) were randomly divided into six groups. Each group of mice was i. v. injected with Dox–Arg-LP or Dox-LP. At 1, 3, 6, 12, 24, and 48 h after administration, blood was collected from the mice in each group. The blood was allowed to clot for 30 min at room temperature and at 4 °C for another 24 h, and the serum was separated through centrifugation at 2000*g* and 4 °C for 20 min. The number of liposomes in the serum was determined by the fluorescence intensity of each sample, using the fluorescence intensity of DiR. The standard calibration curve was obtained through a serial dilution of the original liposomes. A blank serum sample without liposome injection was analyzed to determine the autofluorescence of serum, which was subtracted from the fluorescence intensities of the injected samples during the calculation. The liposomes are represented as the percentage of injected dose per mL of serum.

### Measurement of cell growth inhibition

The experiments were conducted following the procedure of previous works^18,25^ in polycarbonate cell culture inserts which consisted of two compartments separated by a porous membrane (0.4 μm pore size, catalog number 140620, Thermo Scientific Nunc). Colon cancer cells (CT-26) were cultured at the lower chamber at a density of 50 000 cells per well and RAW 264.7 were seeded into the upper chamber in 80 000 cells per well. After 24 h cultured at 37 °C, the medium was exchanged with fresh l-arginine deficient medium, LPS (1 μg mL^−1^) was added to the upper chamber which seeded RAW 264.7 and incubated for 6 h. Then indicated LPs were added in the upper chamber and incubated for 48 h. After separating RAW 264.7 and the cancer cells, the medium was removed, and cells were incubated with fresh medium containing CCK8 reagent for another 4 h. The absorbance was measured at 450 nm by using Infinite M Plex microplate reader.

### *In vivo* evaluation of anticancer effect of Dox–Arg-LP

The anticancer effect of Dox–Arg-LP was investigated *via* the CT-26 colon carcinoma model on 6 weeks BALB/c mice. CT-26 cells (1 × 106) were suspended in 100 μL of Hank's balanced salt solution (Gibco Invitrogen Co), after the mice fur at dorsum was removed, 100 μL cell suspension was subcutaneously injected. The mice were then closely monitored until their tumors reached standard volumes of approximately 100 mm^3^. Tumor volume (*V*) was measured using the following formula: *V* (mm^3^) = (*L* × *W*^2^)/2, where *L* and *W* represent the long and short dimensions of the tumor tissue, respectively. In the next step, animals were divided into six groups (*n* = 6) which were intravenously injected with DPBS, Arg-LP, Dox-LPs (2 or 4 mg kg^−1^ of Dox), or Dox–Arg-LP (2 or 4 mg kg^−1^ of Dox) every two days in total four times. Tumor volume and body weight of mice were calculated and measured up to 15 days post the first injection.

### Statistical analysis

All data are expressed as mean ± SD. Data were evaluated using one-way analysis of variance and statistical analysis was performed *via* the GraphPad Prism software. A value of *p* ≦ 0.05 was considered to be significant.

## Conclusions

The therapeutic efficacy of conventional Dox-LP was enhanced by co-encapsulating Dox with Arg in PEGylated liposomes. The prepared Dox–Arg-LP was determined to be ∼100 nm in diameter and postulated to accumulate in tumor tissue *via* the EPR effect. We showed that liposomes encapsulating Arg, Arg-LP, function as a suitable Arg substrate reservoir for NO production by macrophages, and NO levels increased when Arg-LP was incubated with macrophages. Dox–Arg-LP showed comparable *in vitro* and *in vivo* stability compared with that of Dox-LP. Enhanced Dox cytotoxicity towards cancers cells was observed when Dox–Arg-LP was used in the coculture system. Dox–Arg-LP displayed the strongest antitumor effect *in vivo*. Moreover, 2 mg kg^−1^ Dox dosage in Dox–Arg-LP achieved the same therapeutic effect as twice the dosage of Dox-LP (*i.e.*, 4 mg kg^−1^). Therefore, the administration dosage and frequency can be reduced without loss of antitumor activity, which should improve the quality of life for patients, increase patient compliance and reduce side effects. The synergistic effect observed with this new LP system should facilitate the development of new technology and pharmaceutical innovations for anticancer therapy.

## Conflicts of interest

There are no conflicts to declare.

## Supplementary Material

RA-011-D1RA06514A-s001
