# Supplementary material for: Preparation of a PEGylated liposome that co-encapsulates l-arginine and doxorubicin to achieve a synergistic anticancer effect
Source: RSC Adv. 2021 Oct 21;11(54):34101–6. doi: 10.1039/d1ra06514a (PMC9042383; doi:10.1039/d1ra06514a)
Supplement: RA-011-D1RA06514A-s001 [file RA-011-D1RA06514A-s001.pdf]

Electronic Supplementary Material (ESI) for RSC Advances.
This journal is © The Royal Society of Chemistry 2021

Supplementary information

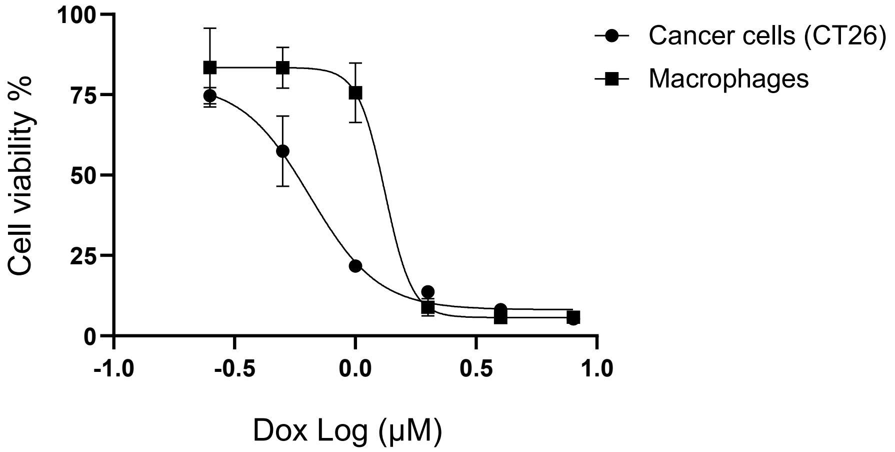

Figure S1. The difference of Dox sensitivity towards cancer cells (CT26) and RAW 264.7 macrophages. Cell viability in increasing concentrations of Dox was determined by CCK8 assay and is expressed as a percentage of the control value. The results are expressed as mean ± SD (n = 3).

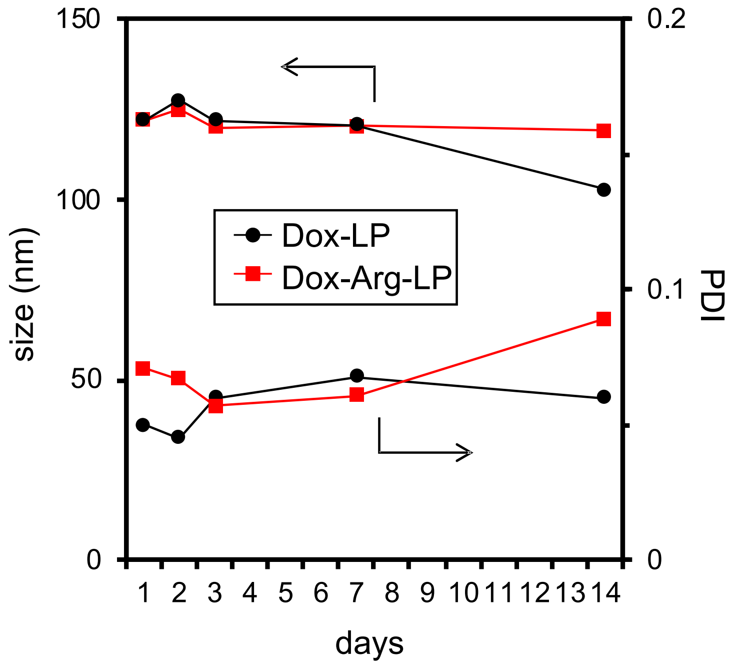

Figure S2. Stability of liposomes during the storage at pH 7.4 and 4 ºC evaluated by size and PDI.
